# Association between Serum Copeptin and Stroke in Rural Areas of Northern China: A Matched Case-Control Study

**DOI:** 10.1155/2018/9316162

**Published:** 2018-10-04

**Authors:** Hao Sun, Donghui Huang, Hailong Wang, Bo Zhou, Xiaomei Wu, Bing Ma, Jingpu Shi

**Affiliations:** ^1^Department of Clinical Epidemiology and Evidence-Based Medicine, Institute of Cardiovascular Diseases, The First Hospital of China Medical University, No. 155 Nanjing North Street, Heping District, Shenyang, Liaoning province 110001, China; ^2^Department of Clinical Epidemiology, Shengjing Hospital of China Medical University, No. 36 Sanhao Street, Heping District, Shenyang, Liaoning province 110004, China

## Abstract

**Background:**

Copeptin has been implicated as an effective prognostic biomarker of stroke outcome; however, few studies have investigated whether copeptin could be used as an etiological factor for stroke or not. The aim of our study was to evaluate the association of serum copeptin with stroke.

**Methods:**

In total, 238 participants including 119 cases (87 ischemic stroke and 32 hemorrhagic stroke) and 119 controls were included in this 1 : 1 matched case-control study. Conditional multivariate logistic regression was conducted to assess the Odds Ratios (*OR*s) and 95% confidence intervals (*CI*); restricted cubic spline in logistic regression model was used to evaluate the dose-response association between serum copeptin and total stroke, ischemic stroke, and hemorrhagic stroke.

**Results:**

The median serum copeptin was 20.90 pmol/L, 20.90 pmol/L, 6.53 pmol/L, and 8.42 pmol/L for total stroke, ischemic stroke, hemorrhagic stroke, and healthy subjects, respectively. The corresponding *OR*s (95% CIs) for the highest compared with the lowest quartile were 1.23 (0.62–2.44) for total stroke, 4.01 (1.47–10.96) for ischemic stroke, and 0.13 (0.22–0.69) for hemorrhagic stroke. No nonlinear dose-response relationship was found between serum copeptin and total stroke (*P*_nonlinear_ = 0.278), ischemic stroke (*P*_nonlinear_ = 0.362), and hemorrhagic stroke (*P*_nonlinear_ = 0.314). Compared with the reference copeptin level, a significantly increasing trend was found between serum copeptin and ischemic stroke (*P*_overall_ = 0.002), and a decreasing trend was found between serum copeptin and hemorrhagic stroke (*P*_overall_ = 0.007).

**Conclusions:**

Elevated serum copeptin levels were positively associated with ischemic stroke and adversely associated with hemorrhagic stroke. Additional prospective studies with larger sample size are needed to confirm the present findings.

## 1. Introduction

Stroke is the second cause of death in elder people (>60 years) and the fifth leading cause of death in people aged 15 to 59 years old throughout the world [1]. In China, stroke has become the leading cause of death [2]; the incidence and prevalence are both increasing rapidly over the 30 years [3]. The National Epidemiology Survey of Stroke in China (NESS-China) has reported there was a north-to-south gradient of stroke burden in China, and the greatest stroke burden was observed in the rural areas of northern regions [3]. Therefore, the rising burden of stroke has built a serious public health problem in China, especially the rural areas of Northern China.

In China, ischemic stroke accounts for approximately 77.8% of total stroke type while the rest 22.2% is subjected to hemorrhagic stroke [3]. Early defining and categorizing of stroke are very important since the managements for ischemic stroke and hemorrhagic stroke are totally different. Currently, computed tomography (CT) and magnetic resonance imaging (MRI) cerebral imaging are most routinely used for confirming the diagnosis of stroke and distinguishing ischemic stroke from hemorrhagic stroke. However, CT and MRI scan are not available easily across all the hospitals, especially in the rural areas of China. Compared with CT and MRI, blood test is a more reliable, rapid, and cost-effective way to differentiate ischemic stroke and hemorrhagic stroke. Therefore, identifying some novel blood biomarkers to distinguish effectively and efficiently ischemic stroke from hemorrhagic stroke might help in the selection of appropriate treatment plans for stroke patients.

Arginine vasopressin (AVP), also termed as antidiuretic hormone, is a key hormone to regulate the osmoregulatory, hemodynamic, endocrinologic, and central nervous effect in human body [4]. However, AVP is hard to detect because it is extremely unstable in both *in vivo* and *ex vivo* environment and has short half-life (5–20 min) [5]. Copeptin, the C-terminal portion of provasopressin (pro-AVP), is released in a 1 : 1 ratio with AVP during precursor processing [6]. Since copeptin has a longer half-life (>90 min) and more stability *ex vivo* than AVP, it could be used as a sensitive surrogate biomarker and a valid measurement in biological fluid samples in routine clinical practice [7, 8]. Several epidemiological studies have demonstrated that copeptin might be used as a prognosis biomarker including predicting neurologic deterioration, risk stratification, and poor outcome (mortality, unfavorable outcome, and recurrence) of stroke [9–13], and the possible mechanism was the copeptin could mirror the hypothalamic-pituitary-adrenal axis as well as the sympathetic nervous system [14], which might promote pathophysiological conditions of stroke. Thus, we hypothesized that copeptin as a novel marker of neuroendocrine might also be associated with stroke risk.

Currently, little evidence was available on the role of copeptin in the patients with stroke and subtypes (ischemic and hemorrhagic) of stroke in China. To evaluate these issues, we investigated the serum copeptin levels among participants in rural areas of Northern China and assessed their relationship with stroke, ischemic stroke, and hemorrhagic stroke.

## 2. Materials and Methods

### 2.1. Study Population

Our study was a large-scale cross-sectional survey, including 11 villages covering various regions (East, South, West, North, and Middle) of Zhangwu County (Liaoning Province, China), an area with high prevalence of hypertension. A total of 6773 eligible subjects aged more than 18 years old were recruited in the survey for the questionnaires, and 6571 subjects agreed to collect blood samples, including 119 stroke patients (87 ischemic stroke and 32 hemorrhagic stroke). The ethics committee of China Medical University approved this study, and all participants offered informed consent.

### 2.2. Case-Control Population

This was a 1 : 1 age- and gender-matched and population-based case-control study. Eligible cases who had been diagnosed stroke were according to their medical records, which were provided by hospitals above county level. The stroke subtypes (ischemic and hemorrhagic) were determined by neurologists based on the clinical assessment and neuroimaging (CT or MRI) according to “The Diagnosis of All Type of Cerebrovascular Disease,” which was adopted by the Fourth National Cerebrovascular Disease Academic Meeting in 1995 [15]. Subjects with the following diseases were excluded: (1) other cerebrovascular diseases (brain tumor, cerebrovascular malformation, or transient ischemic attack), (2) coronary heart disease, and (3) serious chronic diseases (tumor, kidney disease, endocrine disease, and immune system diseases). For each subject in the stroke group, we selected one healthy subject as matched control according to age and gender from this survey. Finally, 119 healthy subjects were included in our study.

### 2.3. Information Collected

The surveys were conducted at village clinics by trained interviewers (village doctors, retired medical professors, and medical students). Structured questionnaires were used to collect the following information: (1) sociodemographic information: name, age, gender, ethnicity, address, and the number of family members; (2) disease information: personal history of hypertension, stroke, and other diseases, family history of stroke; (3) stroke-related factors: smoking status, alcohol consumption, salt intake (g/day), and pickle intake (times/day); and (4) anthropometric information: weight (kg), height (cm), systolic blood pressure (SBP, mmHg), and diastolic blood pressure (DBP, mmHg). Weight, height, and blood pressure were measured twice by using the same type instruments and following standardized protocols.

In our study, hypertension was defined as SBP ≥ 140 mmHg and (or) DBP ≥ 90 mmHg or the use of antihypertensive medicine in the last two weeks [16]. Family history of stroke was defined as the presence of stroke in first degree relatives. Smokers were participants who smoked at least one cigarette per day for more than 1 year, and alcohol drinkers were those who drank alcohol more than three times a week for at least 1 year.

### 2.4. Blood Sample Measurements

Blood samples (5 mL venous blood) were collected from the antecubital vein in the morning after an overnight fast. The samples were centrifuged immediately, and serum samples were transported frozen to the analytical laboratory in Shenyang (Liaoning, China) and stored at −80°C for further analysis. Total cholesterol (TC), triglyceride (TG), and glucose (GLU) were measured according to standard procedures at the Clinical Laboratory, the First Hospital of China Medical University, China. Serum copeptin concentration was assessed with a commercially available enzyme-linked immunosorbent assay (ELISA) kit according to the manufacturer's instructions.

## 3. Statistical Analysis

Means ± standard deviations were used to express the normal distribution of continuous variables; medians and interquartile ranges were used to express the skewed distribution while counts and percentages were used to describe the categorical variable. Differences between groups were calculated by using paired *t*-test, Wilcoxon signed-rank test, Mcnemar's test, and Bowker's test. Moreover, conditional univariate logistic regression was conducted to calculate the Odds Ratios (*OR*s) and 95% confidence intervals (*CI*) of stroke-related factors.

To estimate the association of serum copeptin and stroke, serum copeptin levels were classified into tertiles based on the distribution among controls. The lowest tertile was used as reference level, and conditional univariate logistic regression and conditional multivariate logistic regression were conducted to assess the *OR* for the second and third tertiles. For the conditional multivariate logistic regression, covariates were selected and adjusted if they might be a confounder factor between serum copeptin and stroke (*P* < 0.05 in the conditional univariate logistic regression). The covariates included in the final model were as follows: (1) total stroke: personal history of hypertension, smoking status, and pickle intake; (2) ischemic stroke: BMI, personal history of hypertension, smoking status, and pickle intake; and (3) hemorrhagic stroke: none. Moreover, tests for linear trend were conducted by including serum copeptin as a continuous variable in the models. Besides, restricted cubic spline function with four knots (5^th^, 35^th^, 65^th^, and 95^th^) with reference copeptin level (25.9 pmol/L) [17, 18] in the logistic regression model was used to evaluate the dose-response association between serum copeptin and stroke, and the nonlinear effect and overall effect of the relationship were explored with the likelihood ratio test [19].

All analyses were carried out using the SAS software (version 9.1.3; SAS Institute, Cary, NC). And all statistical tests were based on two-sided probability.

## 4. Results

The characteristics of cases, including cases of total stroke, ischemic stroke, and hemorrhagic stroke, and their matched controls are presented in Table 1. In total, 119 stroke cases (including 87 ischemic stroke and 32 hemorrhagic stroke) and 119 controls were included in this study. The mean age was 62 years for stroke cases, 61 years for ischemic stroke cases, and 63 years for hemorrhagic stroke cases. Significant differences were found between stroke cases and controls for personal history of hypertension, smoking, and pickle intake. For ischemic stroke, BMI, personal history of hypertension, smoking status, and pickle intake were found to have significant difference between two groups. And for hemorrhagic stroke, no significant difference was found between groups.

The median levels of serum copeptin in cases and controls were also described in Table 1. The copeptin concentration was higher among stroke cases compared with that among control participants (20.90 *vs*. 8.42 pmol/L; *P* < 0.001). And in analyses stratified by stroke subtype, higher copeptin level was observed for ischemic stroke cases (20.90 *vs*. 9.55 pmol/L; *P* < 0.001) and lower copeptin level for hemorrhagic stroke cases (6.53 *vs*. 10.79 pmol/L; *P* < 0.001).

The associations between serum copeptin and total stroke, ischemic stroke, and hemorrhagic stroke by tertiles of copeptin levels are presented in Table 2. A positive association was observed between serum copeptin and total stroke (*OR*_rude_ = 1.45, 95% CI: 0.78–2.69 for tertile 3 *vs*. tertile 1; *OR*_adjusted_ = 1.23, 95% CI: 0.62–2.44 for tertile 3 *vs*. tertile 1); however, no statistical significance was found for this increasing trend (*P*_trend_ = 0.052). For ischemic stroke, a positive association was observed between serum copeptin and ischemic stroke (*P*_trend_ = 0.002). Compared with the lowest tertile (<6.478 pmol/L), the *OR* in the highest tertile (>13.599 pmol/L) was 3.99 (*OR*_rude_ = 3.99, 95% CI: 1.66–9.57); the association existed even after being adjusted for potential confounders (*OR*_adjusted_ = 4.01, 95% CI: 1.47–10.96). In contrast, we found a significantly adverse association between serum copeptin level and hemorrhagic stroke. Compared with the lowest copeptin level, the risk of hemorrhagic stroke was significantly lower in the two higher tertiles of copeptin level (tertile 2: *OR* = 0.44, 95% CI: 0.13–1.34; tertile 3: *OR* = 0.13, 95% CI: 0.22–0.69; *P*_trend_ = 0.011).

**Figure 1:**
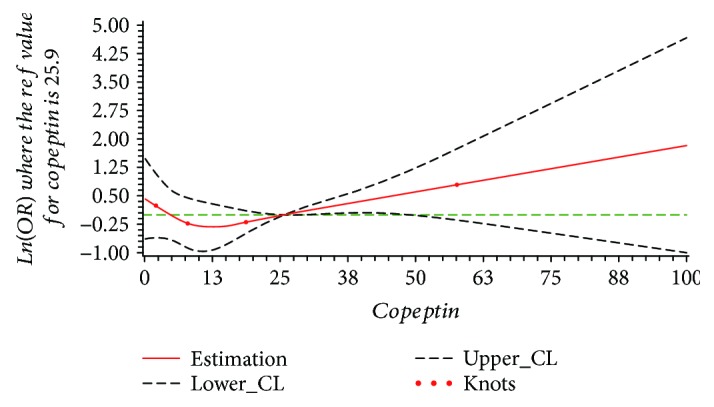
The plot of dose-response relationship between serum copeptin and total stroke (*P*_nonlinear_ = 0.278, *P*_overall_ = 0.139; adjusted by personal history of hypertension, smoking status, and pickle intake).

**Figure 2:**
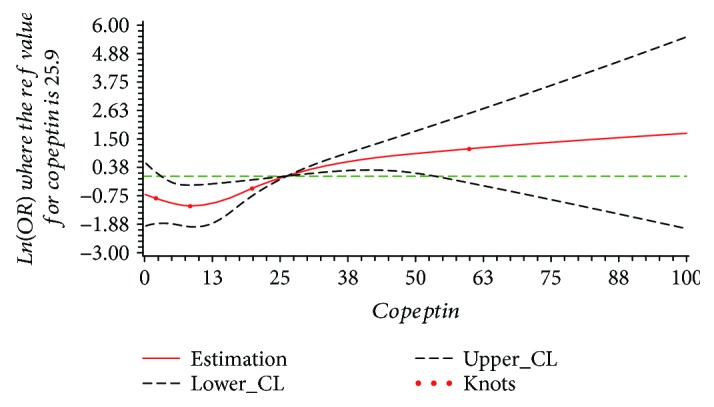
The plot of dose-response relationship between serum copeptin and ischemic stroke (*P*_nonlinear_ = 0.362, *P*_overall_ = 0.002; adjusted by BMI, personal history of hypertension, smoking status, and pickle intake).

**Figure 3:**
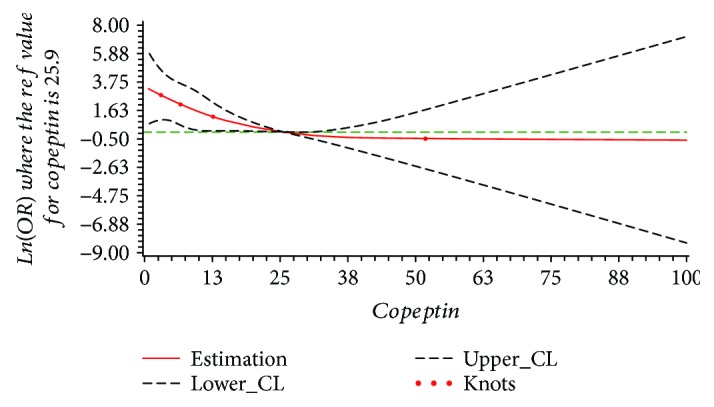
The plot of dose-response relationship between serum copeptin and hemorrhagic stroke (*P*_nonlinear_ = 0.314, *P*_overall_ = 0.007).

The results of dose-response relationship between serum copeptin and total stroke, ischemic stroke, and hemorrhagic stroke are shown in Table 3. In the restricted cubic spline function model with nonlinear terms, no nonlinear dose-response relationship was found between serum copeptin and total stroke (*P*_nonlinear_ = 0.278). Compared with the reference copeptin level (25.9 pmol/L), the prevalence of stroke increased with serum level of copeptin in a dose-response manner; however, no statistical significance was found for this increasing trend (*P*_overall_ = 0.139), as shown in Figure 1. For the subtype of stroke, no nonlinear dose-response relationship was found in the ischemic stroke group or hemorrhagic stroke group (ischemic stroke: *P*_nonlinear_ = 0.362; hemorrhagic stroke: *P*_nonlinear_ = 0.314). The results of dose-response relationship analysis also show that with the serum copeptin concentration increasing, the prevalence of ischemic stroke increased (*P*_overall_ = 0.002), while the prevalence of hemorrhagic stroke decreased (*P*_overall_ = 0.007), just as shown in Figures 2 and 3. The results also reported that in every 1 pmol/L serum copeptin increase, the prevalence of ischemic stroke increased 5% and the prevalence of hemorrhagic stroke decreased 8%.

## 5. Discussion

In this 1 : 1 matched case-control study among participants in rural areas of Northern China, we found that the increased levels of serum copeptin were significantly associated with higher risk of ischemic stroke and lower risk of hemorrhagic stroke in a dose-response manner. The prevalence of ischemic stroke increased 5% and the prevalence of hemorrhagic stroke decreased 8% for every 1 pmol/L serum copeptin increase. A positive association was observed between serum copeptin and total stroke; however, no statistical significance was found for this increasing trend. Our results suggested that copeptin might be a meaningful biomarker to discriminate the subtype of stroke and to play a potential role of the AVP system in the pathophysiology of both ischemic stroke and hemorrhagic stroke.

Our study reported the dose-response association between serum copeptin and stroke in Chinese population. To our knowledge, only three articles have reported the relationship between copeptin and stroke risk [20–22], and the results were inconsistent. A population-based nested case-control study in the United States (The Northern Manhattan Study, NOMAS) found no association of serum copeptin with ischemic stroke risk [20]; another hospital-based cross-sectional study reported that plasma copeptin levels in patients with cerebral infarction, intracranial hemorrhage, and subarachnoid hemorrhage were significantly higher than that in healthy volunteers [22]. The British Regional Heart Study was a prospective study with older male participants with or without diabetes; the results demonstrated that higher serum copeptin level was independently associated with an increased risk of stroke among men with diabetes but not men without diabetes [21]. Therefore, our study might offer a robust evidence to fully evaluate the causal association between serum copeptin and stroke considering the dose-response relationship and the subtype of stroke among general population.

Since there are limited numbers of etiological studies and experimental studies involving copeptin and stroke, the possible mechanisms underlying the relationship between copeptin and ischemic stroke or hemorrhagic stroke remain unclear. According to the previous studies of copeptin, two possible mechanisms about copeptin and ischemic stroke were hypothesized. First, atherosclerotic disease caused the large majority of ischemic strokes [23]; copeptin as one of the biomarkers to reflect intima-media thickness and number of carotid plaques in carotid atherosclerosis might provide some evidences about the progression of ischemic stroke [24]. Second, the antidiuretic effect of vasopressin was mediated by vasopressin 2 receptors, and this effect might increase the microalbuminuria excretion [25], suggesting copeptin might play a role for the AVP system in the development of albuminuria. A meta-analysis had proposed that microalbuminuria strongly and independently associated with the incidence of stroke and atherosclerosis might be the pathological link between microalbuminuria and stroke [26]. Regarding the hemorrhagic stroke, our results presented that higher serum copeptin level was adversely associated with hemorrhagic stroke, which was beyond our expectations. However, Faraci's study proved that endogenous AVP might play a role during hypoxia and intracranial hypertension by a negative feedback mechanism to reduce blood blow to the choroid plexus, which indicated that higher serum copeptin might be a protective factor for hemorrhagic stroke biologically plausible. Since the copeptin mostly played an indirect role in the progression of stroke in the possible mechanisms we mentioned above, cellular and molecular experiments need to be conducted in the future to explore the direct mechanisms between copeptin and ischemic stroke or hemorrhagic stroke.

In clinical practice, several studies had proved that copeptin could be applied to predict severity and outcome of stroke [9–13]. Our findings also indicated that copeptin might be used as an auxiliary biomarker to differentiate ischemic stroke and hemorrhagic stroke in clinical application, which is crucial for the immediate application of the right therapy to patients. Besides, just like the copeptin utility on suspected acute myocardial infarction, copeptin could also be used as one of combined biomarker that might improve accuracy for the early identification of different subtype of stroke. Furthermore, considering the lack of clinical characteristics that clearly differentiate stroke and stroke mimics, studies of Wendt et al. and von Recum et al. had explored whether copeptin could also be used as a potential biomarker to discriminate stroke and stroke mimics in prehospital stroke setting [27, 28]. Even though the results were not significant, these two studies throw light on the value of copeptin to discover noninvasive tests that aim to quickly distinguish stroke and stroke mimics and offer valuable recommendations to further studies.

Some limitations of our study must be considered. First, considering the budgets and efficiency, our study was designed as a case-control study, which was more likely to generate bias than a prospective cohort study design, and the causality of copeptin and stroke could not be inferred from the observed association. Second, the possibility of a type I error might occur in our study due to relatively small sample size; therefore, additional studies with a larger sample size are needed to confirm our findings. Third, although we considered some potential confounding factors, such as age, gender, family history of stroke, and personal history of hypertension, we could not exclude the possibility of residual confounding from inadequately measured covariates or confounders.

## 6. Conclusion

In summary, in this 1 : 1 age- and gender-matched and population-based case-control study, we observed that elevated serum copeptin levels were positively associated with ischemic stroke, while elevated serum copeptin levels were adversely associated with hemorrhagic stroke; no significant association was found between serum copeptin level and total stroke. Our results suggested a role of copeptin in the pathophysiological conditions of stroke; however, the confirmation of our findings in other prospective studies and further considerations of potential mechanisms are still needed.

## Figures and Tables

**Table 1:** Characteristics of cases of total stroke, ischemic stroke, and hemorrhagic stroke and their matched controls^a^.

	Total stroke				Ischemic stroke				Hemorrhagic stroke			
	Case (*n* = 119)	Control (*n* = 119)	*P* ^b^	OR (95% CI)^c^	Case (*n* = 87)	Control (*n* = 87)	*P* ^b^	OR (95% CI)^c^	Case (*n* = 32)	Control (*n* = 32)	*P* ^b^	OR (95% CI)^c^
Age (years)	62 (53–69)	62 (53–69)	—	—	61 (52–70)	61 (52–70)	—	—	63 (56–68)	63 (56–68)	—	—
Gender (male; n, %)	146 (61.34%)	146 (61.34%)	—	—	56 (64.4%)	56 (64.4%)	—	—	17 (53.1%)	17 (53.1%)	—	—
BMI (kg/m^2^)	24.05 ± 3.85	24.84 ± 3.44	0.105	0.94 (0.88–1.01)	23.68 ± 3.74	25.02 ± 3.38	0.015	0.90 (0.82–0.98)	25.06 ± 4.03	24.37 ± 3.60	0.490	1.05 (0.92–1.19)
Personal history of hypertension (n, %)	92 (77.31%)	76 (63.87%)	0.014	2.23 (1.16–4.29)	68 (78.2%)	54 (62.1%)	0.016	2.40 (1.15–5.02)	24 (75.0%)	22 (68.8%)	0.480	1.67 (0.40–6.97)
Family history of stroke (n, %)	27 (22.69)	24 (20.17%)	0.631	1.17 (0.62–2.19)	18 (20.7%)	19 (21.8%)	0.847	0.93 (0.44–1.98)	9 (28.1%)	5 (15.6%)	0.248	2.00 (0.60–6.64)
Smoking (n, %)	65 (54.62%)	45 (37.82%)	0.002	2.82 (1.42–5.61)	53 (60.9%)	36 (41.4%)	0.003	3.13 (1.41–6.93)	12 (37.5%)	9 (28.1%)	0.317	2.00 (0.50–8.00)
Alcohol drinking (n, %)	39 (32.77%)	42 (35.29%)	0.622	0.85 (0.45–1.62)	30 (34.5%)	33 (37.9%)	0.564	0.80 (0.37–1.71)	9 (28.1%)	9 (28.1%)	1.000	1.00 (0.29–3.45)
Salt intake (≥180 g/month; n, %)	107 (88.92%)	104 (87.39%)	0.513	1.33 (0.56–3.16)	81 (93.1%)	78 (89.7%)	0.405	1.60 (0.52–4.89)	26 (81.3%)	26 (81.3%)	1.000	1.00 (0.25–4.00)
Pickle intake (times/day)			0.198				0.350				0.936	
0	30 (25.21%)	35 (29.41%)	—	1.00 (reference)	22 (25.3%)	23 (26.4%)	—	1.00 (reference)	8 (25%)	12 (37.5%)	—	1.00 (reference)
1	23 (19.33%)	28 (23.53%)	—	0.79 (0.43–1.45)	17 (19.5%)	20 (23.0%)	—	0.83 (0.42–1.65)	6 (18.8%)	8 (25%)	—	0.67 (0.19–2.36)
2	7 (5.88%)	15 (12.61%)	—	0.39 (0.14–1.08)	4 (4.6%)	12 (13.8%)	—	0.27 (0.08–0.99)	3 (9.4%)	3 (9.4%)	—	1.00 (0.14–7.10)
3	59 (49.58%)	41 (34.45%)	—	1.78 (1.07–2.97)	44 (50.6%)	32 (36.8%)	—	1.63 (0.92–2.89)	15 (46.9%)	9 (28.1%)	—	2.50 (0.78–7.97)
TC (mmol/L)	5.24 (4.48–5.75)	5.08 (4.39–5.49)	0.168	1.22 (0.92–1.61)	5.30 (4.49–5.79)	5.08 (4.38–5.40)	0.122	1.38 (0.96–2.00)	5.21 (4.47–5.63)	5.26 (4.48–5.86)	0.758	0.94 (0.75–1.18)
TG (mmol/L)	1.50 (1.02–2.38)	1.40 (0.98–2.07)	0.694	1.00 (0.82–1.20)	1.50 (1.03–2.42)	1.47 (1.01-2.11)	0.929	0.94 (0.75–1.18)	1.59 (1.07–2.35)	1.35 (0.97–1.96)	0.454	1.16 (0.79–1.68)
GLU (mmol/L)	5.00 (4.30–5.30)	4.60 (4.20–5.20)	0.107	1.15 (0.94–1.40)	5.07 (4.50–5.30)	4.70 (4.20–5.10)	0.196	1.16 (0.92–1.47)	4.80 (4.20–5.25)	4.55 (4.20–5.30)	0.388	1.11 (0.75–1.63)
Copeptin (pmol/L)	20.90 (8.65–37.40)	8.42 (5.45–15.33)	<0.001	1.02 (1.00-1.03)	20.90 (9.76–37.60)	9.55 (5.66–16.70)	<0.001	1.05 (1.02–1.08)	6.53 (4.94–9.47)	10.79 (7.28–28.87)	<0.001	0.92 (0.86–0.98)

^a^Adjusted for age and gender at sample collection. ^b^Continuous variables were compared by using paired *t*-test and Wilcoxon signed-rank test; categorical variables were compared by using Mcnemar's test and Bowker's test. ^c^The ORs and 95% CIs were derived from conditional univariate logistic regression.

**Table 2:** Association between the level of serum copeptin and total stroke, ischemic stroke, and hemorrhagic stroke.

	Level of serum copeptin		
	Tertile 1	Tertile 2	Tertile 3
Total stroke (*n* = 119 sets)			
Copeptin range (pmol/L)	<7.256	7.256~16.440	>16.440
Number of cases/controls	37/40	28/39	54/40
*OR* (95% CI)	1.00 (reference)	0.77 (0.39–1.54)	1.45 (0.78–2.69)
*OR* (95% CI)^a^	1.00 (reference)	0.70 (0.33–1.49)	1.23 (0.62–2.44)
*P*_trend_^a^	0.052		
Ischemic stroke (*n* = 87 sets)			
Copeptin range (pmol/L)	<6.478	6.478~13.599	>13.599
Number of cases/controls	17/29	15/30	55/28
*OR* (95% CI)	1.00 (reference)	0.82 (0.33–2.05)	3.99 (1.66–9.57)
*OR* (95% CI)^b^	1.00 (reference)	0.79 (0.28–2.21)	4.01 (1.47–10.96)
*P*_trend_^b^	0.002		
Hemorrhagic stroke (*n* = 32 sets)			
Copeptin range (pmol/L)	<8.425	8.425~26.019	>26.019
Number of cases/controls	19/10	11/12	2/10
*OR* (95% CI)	1.00 (reference)	0.44 (0.13–1.43)	0.13 (0.22–0.69)
*P*_trend_	0.011		

^a^The *OR* was adjusted by personal history of hypertension, smoking status, and pickle intake. ^b^The *OR* was adjusted by BMI, personal history of hypertension, smoking status, and pickle intake. *P*_trend_: *P* value for trend was derived from conditional logistic regression in which serum copeptin levels were used as continuous variables and adjusted by related factors.

**Table 3:** Dose-response relationship between serum copeptin and total stroke, ischemic stroke, and hemorrhagic stroke.

Serum copeptin (pmol/L)	Total stroke^a^		Ischemic stroke^b^		Hemorrhagic stroke	
	*OR*	95% CI	*OR*	95% CI	*OR*	95% CI
5.9	0.92	0.48–1.74	0.34	0.15–0.75	9.17	1.96–43.01
15.9	0.76	0.48–1.20	0.44	0.25–0.80	2.21	1.11–4.38
25.9	1.00 (reference)		1.00 (reference)		1.00 (reference)	
35.9	1.30	1.04–1.62	1.70	1.26–2.30	0.69	0.37–1.26
45.9	1.67	1.03–2.70	2.34	1.24–4.42	0.61	0.13–2.87
1 pmol/L increment	1.02	1.00-1.03	1.05	1.02–1.08	0.92	0.86–0.98
*P*_nonlinear_	0.278		0.362		0.314	
*P*_overall_	0.139		0.002		0.007	

^a^The *OR* was adjusted by personal history of hypertension, smoking status, and pickle intake. ^b^The *OR* was adjusted by BMI, personal history of hypertension, smoking status, and pickle intake.

## Data Availability

The data used to support the findings of this study are available from the corresponding author upon request.
